# RETRACTED: Li et al. Blockade of Y177 and Nuclear Translocation of Bcr-Abl Inhibits Proliferation and Promotes Apoptosis in Chronic Myeloid Leukemia Cells. *Int. J. Mol. Sci.* 2017, *18*, 537

**DOI:** 10.3390/ijms27188230

**Published:** 2026-09-16

**Authors:** Qianyin Li, Zhenglan Huang, Miao Gao, Weixi Cao, Qin Xiao, Hongwei Luo, Wenli Feng

**Affiliations:** 1 Department of Clinical Hematology, Key Laboratory of Laboratory Medical Diagnostics Designated by the Ministry of Education, Chongqing Medical University, Chongqing 400016, Chinagaomiao_1@163.com (M.G.); weixicqmu@163.com (W.C.);; 2 Department of Hematology, The First Affiliated Hospital of Chongqing Medical University, Chongqing 400016, China

The Journal retracts the article “Blockade of Y177 and Nuclear Translocation of Bcr-Abl Inhibits Proliferation and Promotes Apoptosis in Chronic Myeloid Leukemia Cells” [1] cited above.

Following publication, concerns were brought to the attention of the Editorial Office regarding the integrity of the figures presented in this article [1].

In accordance with standard journal procedures, the Editorial Office and Editorial Board conducted an investigation that identified indications of inappropriate image editing and duplication within Figures 4 and 5, and apparent image duplication between Figures 4D and 5B,C presented in this article. Although the authors cooperated with the investigation, the explanations and supporting materials provided were not deemed sufficient by the Editorial Board to resolve the identified concerns. As a consequence, the Editorial Board has lost confidence in the reliability of the reported findings and has decided to retract this publication in accordance with MDPI’s retraction policy.

This retraction was approved by the Editor-in-Chief of the journal *IJMS*.

The authors have been informed of this retraction and have indicated their disagreement with this decision.
